# UPLC-ESI/MS^n^ metabolic profiling of *Cedrela odorata* L. and *Toona ciliata* M. Roem and *in vitro* investigation of their anti-diabetic activity supported with molecular docking studies

**DOI:** 10.3389/fchem.2024.1462309

**Published:** 2024-11-15

**Authors:** Heba A. S. El-Nashar, Ayman M. Al-Qaaneh, Md. Shimul Bhuia, Raihan Chowdhury, Mostafa A. Abdel-Maksoud, Hossam Ebaid, Abdul Malik, Muhammad Torequl Islam, Mohammed Aufy, Esraa A. Elhawary

**Affiliations:** ^1^ Department of Pharmacognosy, Faculty of Pharmacy, Ain Shams University, Cairo, Egypt; ^2^ Department of Allied Health Sciences, Al-Balqa Applied University (BAU), Al-Salt, Jordan; ^3^ Department of Pharmaceutical Technology, Faculty of Pharmacy, Jordan University of Science and Technology (JUST), Irbid, Jordan; ^4^ Bioinformatics and Drug Innovation Laboratory, BioLuster Research Center, Dhaka, Bangladesh; ^5^ Department of Pharmacy, Bangabandhu Sheikh Mujibur Rahman Science and Technology, University, Gopalganj, Bangladesh; ^6^ Botany and Microbiology Department, College of Science, King Saud University, Riyadh, Saudi Arabia; ^7^ Department of Zoology, College of Science, King Saud University, Riyadh, Saudi Arabia; ^8^ Department of Pharmaceutics, College of Pharmacy, King Saud University, Riyadh, Saudi Arabia; ^9^ Pharmacy Discipline, Khulna University, Khulna, Bangladesh; ^10^ Department of Pharmaceutical Sciences, Division of Pharmacology and Toxicology, University of Vienna, Vienna, Austria

**Keywords:** *Cedrela*, ultra-performance liquid chromatography-electrospray ionization-mass spectrometry, anti-diabetic, α-amylase, α-glucosidase, phenylpropanoids

## Abstract

**Introduction:**

The genus *Cedrela* is one of the phytochemically rich genera of the family Meliaceae. In this study, two *Cedrela* species, namely, *Cedrela odorata* and *Toona ciliata* M. Roem (formerly *Cedrela toona*), were selected for in-depth phytochemical profiling with the aid of UPLC-ESI/MS^n^ analysis followed by evaluation of their anti-diabetic potential through assessment of *in vitro α*-amylase and *α*-glucosidase inhibitory effects, alongside the molecular docking studies on these target enzymes.

**Materials and methods:**

UPLC-ESI/MS^n^ technique was applied to tentatively identify the extracts. The anti-diabetic properties were assessed using BioVision *α*-amylase and *α*-glucosidase inhibitor screening kits. Further, the molecular docking studies utilized PyRx® and Discovery Studio software.

**Results and discussion:**

The UPLC-ESI/MS^n^ analysis led to the identification and quantification of 55 metabolites with their fragmentation patterns for the first time for these two species. Flavonoids represented the main identified class, followed by phenylpropanoids, terpenes, tannins, and others. The two species showed potent enzyme inhibition, where *C. odorata* and *C. toona* significantly inhibited *α*-amylase (IC_50_ = 4.83 ± 0.01 and 3.50 ± 0.03 μg/mL) compared to pioglitazone (IC_50_ = 2.17 ± 0.23 μg/mL), while their *α*-glycosidase inhibitory properties were also potent with (IC_50_ = 7.17 ± 0.01 and 6.50 ± 0.69 μg/mL), respectively, compared to acarbose (IC_50_ = 4.83 ± 1.02 μg/mL). The enzyme inhibitory activities were further confirmed by *in silico* molecular docking of the main identified components with the respective binding sockets in both *α*-amylase and *α*-glycosidase enzymes.

**Conclusion:**

These promising results could pave the way for a novel discovery of natural phytoconstituents with potent anti-diabetic activity.

## Graphical Abstract

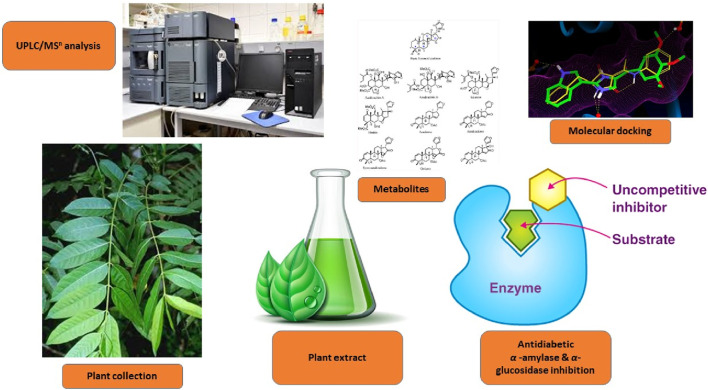

## 1 Introduction

*Diabetes mellitus* (DM) is a chronic metabolic disease with increasing prevalence and incidence worldwide at an alarming rate (Kaur et al., 2018; El‐Nashar et al., 2024a; Todirascu-Ciornea et al., 2019). The most common type of DM is type-2 diabetes (non-insulin-dependent), which mostly affect adults and accounts for 90% of all diabetes cases (Mukhtar et al., 2020; Abdelghffar et al., 2022). Various oral commercial anti-diabetic drugs like biguanides, meglitinide, sulfonylureas, thiazolidinedione, dipeptidyl peptidase-4 (DPP-4) inhibitors, sodium–glucose cotransporter, and carbohydrate-hydrolyzing enzyme inhibitors have been implemented to control postprandial hyperglycemia (Chaudhury et al., 2017; El‐Nashar et al., 2023). Some of the most effective anti-diabetic drugs are *α*-glucosidase and *α*-amylase inhibitors used for reducing postprandial hyperglycemia (Derosa and Maffioli, 2012; Saber et al., 2023). The available marketable inhibitors such as acarbose, voglibose, and miglitol competitively inhibit these metabolic enzymes, and thus delay digestion, leading to reduction of carbohydrate absorption and thus constraining postprandial hyperglycemia (Derosa and Maffioli, 2012). These drugs are administrated prior to consumption of complex carbohydrate-rich meals, which reduces the glycated hemoglobin (Hb_A1c_) levels, but these drugs suffer from frequent gastrointestinal tract (GIT)-related side effects (Godbout and Chiasson, 2007). Accordingly, several scientists are devoted to the discovery of novel *α*-glucosidase and/or *α*-amylase inhibitors with fewer or no undesired effects from natural sources (Riyaphan et al., 2021). Nowadays, plant-based medicines and functional foods have generated a renewed interest for the prevention and cure of diabetes, given their few or no side effects (Tundis et al., 2010; Rabie et al., 2023). The plant kingdom offers rich arrays of natural bioactive hypoglycemic agents (Hung et al., 2012; El-Nashar et al., 2021a). In the past few decades, over 1,200 plant species have been empirically used as hypoglycemic agents worldwide (Ervina, 2020; Jamaddar et al., 2023; El-Nashar et al., 2022). Consequently, the natural inhibitors of *α*-glucosidase and *α*-amylase from plant sources are considered an attractive strategy for treating hyperglycemia (El-Nashar et al., 2024a; El-Nashar et al., 2021b).

*C. odorata* is a fast-growing perennial tree with paripinnate leaves, belonging to the family Meliaceae (Cavers et al., 2013). This species originated from Pacific and Atlantic Central America, the Antilles, South America, both east and west of the Andes, central and eastern coastal Brazil, and northern Argentina. It is a monecious tree, pollinated by small insects with small, wind-dispersed seeds. It grows up to 800 m, but in Ecuador, some trees grow up to 1,500 m (Galván-Hernández et al., 2018). Even though it grows in both evergreen rain forest and drier forest, it can thrive in dry ecological habitats such as deciduous habitat, and buds are protected by scaly leaves (Finch et al., 2022). The fruit takes a long time for maturation in the dry season (Cavers et al., 2013). In folk medicine, the stem bark infusion of *C. odorata* was used for the treatment of fever, hemorrhage, inflammation, and digestive diseases, including diarrhea, vomiting, and indigestion, in South America. The bark decoction was used for as malarial treatment and for fever in Africa. The family Meliaceae and especially genus *Cedrela* are rich in limonoids, alkaloids, and polyphenols such as lignans and proanthocyanidins (Muñoz Camero et al., 2018).

*Toona ciliata* M. Roem (formerly *Cedrela toona* Roxb.) is a medium- to large-sized deciduous tree with a brown-to-gray scaly bark. Leaves are 15–45 cm long, usually paripinnate, but sometimes with a terminal leaflet; leaflets are mostly 8–20 cm, ovate in shape, 4–15 cm long, 15–50 mm wide, apex acuminate, base strongly asymmetric, margins entire, mostly glabrous, the petiole is 4–11 cm long, and petiolules are 5–12 mm long. The penicles are 20–40 cm long. The petals are 5–6 mm long and white in color. The capsules are ellipsoid, 10–20 mm long, 6–8 mm diameter; seeds are winged at both ends. Traditionally, the bark is reported to be used as astringent, anti-dysenteric, and anti-periodic. Flowers are emmenagogue, and the leaf is spasmolytic, hypoglycemic, and anti-protozoal (Shah and Patel, 2021).

The current study was directed to comparatively analyze the phytoconstituents of the 80% methanol leaf extracts of *Cedrela odorata* and *Toona ciliata* M. Roem via the UPLC-ESI/MS^n^ technique and investigate *α*-amylase and *α*-glucosidase inhibitory properties. Moreover, molecular docking experiments were carried out to assess the binding affinities of plant extract components with the targeted enzymes.

## 2 Material and methods

### 2.1 Plant material collection

The fresh leaves of *Cedrela odorata* and *Toona ciliata* were obtained from The Animal Zoo Garden, Dokki, Giza, Egypt (30°01′16.99″N 31°12′30.01″E) in February 2023. The leaves of each species were taxonomically identified by Mrs. Tereize Labib, the taxonomy specialist at El-Orman Botanical Garden, Giza, Egypt. The voucher specimens (PHG-P-CO-482 and PHG-P-CT-481) have been kept for *Cedrela odorata* and *Toona ciliata*, respectively, in the Herbarium of the Pharmacognosy Department, Faculty of Pharmacy, Ain Shams University, Cairo, Egypt.

### 2.2 Preparation of plant extracts

The fresh leaves of each species (0.5 kg) were finely cut and soaked in 80% aqueous methanol (5 L, BioChem. Comp., Egypt) by percolation at room temperature till depletion. Then, the extracts were filtrated and concentrated under reduced pressure using a rotavapor (Buchi, R-300) to yield completely dry extracts of *Cedrela odorata* (10.31 gm) and *Toona ciliata* (8.06 gm).

### 2.3 Ultra-performance liquid chromatography–electrospray ionization-mass spectrometry (UPLC-ESI/MS^n^) analysis

UPLC-ESI/MS^n^ in both positive and negative ion acquisition modes were carried out according to the method adopted from Elhawary et al. (2021).

### 2.4 Assessment of *in vitro* anti-diabetic activities

#### 2.4.1 *α*-Amylase inhibition assay

The *α*-amylase inhibitory activities of the tested *Cedrela* extracts were determined according to the standard published procedure with minor amendments (Kazeem et al., 2013). The enzyme solution was prepared by dissolving *α*-amylase in 20 mM phosphate buffer (pH = 6.9) at a concentration of 0.50 mg/mL. Then, 1 mL of different concentrations of the tested extract (0.01–100 μg/mL) was mixed with 1 mL of enzyme solution and allowed to sit at room temperature for 10 min. After incubation, 1 mL of 0.50% starch solution was included to the mixture and further incubated at room temperature for 10 min. The reaction was then ended by addition of 3,5-dinitrosalicylic acid (2 mL) and then heating the reaction mixture in a boiling water bath for 5 min. After cooling, the absorbance of the mixture was colorimetrically determined at 565 nm. Pioglitazone was used as a standard drug. The inhibition percentage was calculated using the following formula.

$$\%\text{ inhibition}=\left(1-\text{As}/\text{Ac}\right)\times 100,$$

whereAs = the absorbance of the tested extract andAc = the absorbance of the control reaction (containing all reagents except the test sample).

The IC_50_ value was specified as the concentration of the plant extract to inhibit 50% of *α*-amylase activity under the experiment conditions.

#### 2.4.2 *α*-Glucosidase inhibition assay

The *α*-glucosidase inhibitory activities of the plant extracts were determined based on the previously described technique using the BioVision *α*-glucosidase inhibitor screening kit (K938-100) (El-Nashar et al., 2021b). The tested plant extract (10 µL) was mixed with the same volume of glutathione and *α*-glucosidase solution (in phosphate buffer (pH = 6.8), and 4-nitrophenyl-*α*-D-glucopyranoside (10 µL) in a 96-well microplate and incubated for 15–20 min at 25°C. Likewise, the blank solution was prepared by adding the plant extract to all used reaction reagents lacking α-glucosidase solution. The principle of the reaction is based on the ability of an active α-glucosidase to cleave a synthetic substrate (4-nitrophenyl-*α*-D-glucopyranoside), into a chromophore (p-nitrophenol; OD = 410 nm). Acarbose was used as a standard drug. The reaction was then stopped upon addition of 50 µL of sodium carbonate (0.2 M). The absorbance of the tested extract and blank was assessed at 410 nm. The absorbance of the blank was subtracted from the values of the tested extract, and the results were stated as IC_50_.

### 2.5 *In silico* molecular docking experiments

#### 2.5.1 Ligand preparation

Data of most of the ligands were downloaded from the PubChem^®^ chemical database, while some were drawn using ChemDraw^®^ Professional software. The structures of the chemical compounds were then converted into 3D format and their energy minimized using Chem3D^®^ Pro software, employing the MM2 Allinger’s force field method. All ligands were imported as SDF files for conducting the molecular docking study (Bhuia et al., 2023).

#### 2.5.2 Target enzyme collection and preparation

Based on the literature review, it is known that *alpha*-amylase and *alpha*-glucosidase play crucial roles in the pathophysiology of diabetic disorders. The information of the targeted enzymes, *alpha*-amylase (PDB ID: 4GQR) and *alpha*-glucosidase (PDB ID: 3TOP), was obtained from the RCSB Protein Data Bank. Subsequently, the targeted enzymes underwent optimization by removal of water molecules, co-crystal ligands, and unwanted protein chains using Discovery Studio^®^ software (Afroz et al., 2024). Following the optimization process, the targets were subjected to energy minimization utilizing the Swiss PDB Viewer^®^ software package, employing the GROMOS96 force field method, and saved in PDB file format (Chowdhury et al., 2023).

#### 2.5.3 Molecular docking

Molecular docking was conducted on the selected ligands with the *alpha*-amylase and *alpha*-glucosidase enzymes using PyRx^®^ software. In the docking process, the target enzyme was uploaded and converted into macromolecules. Subsequently, the ligand was loaded and converted into the PDBQT format. We performed blind docking, setting the grid box to its maximum along the X, Y, and Z axes. The docking results of the study were saved in the CSV file format, and the best pose was extracted in the PDB file format. The interactions between the ligand and enzyme were visualized using Discovery Studio^®^ software (Chowdhury et al., 2024). Additionally, non-bond interactions of the ligand and enzyme were visualized using Discovery Studio software (Chowdhury et al., 2024). Additionally, non-bond interactions of the ligand and enzyme were recorded.

### 2.6 Statistical analysis

The assays were performed in triplicates, and the obtained values are stated as mean ± SD. For the *in vitro* investigation of *α*-amylase and *α*-glucosidase inhibitory properties, the IC_50_ was obtained from the graph plots of the dose–response curves at each oil concentration via Graph Pad Prism^®^ software (San Diego, CA, United States). The IC_50_ is the concentration of the extract required to inhibit 50% of the tested enzyme activity under the applied assay conditions.

## 3 Results and discussion

### 3.1 UPLC-ESI/MS^n^ metabolic profiling of the 80% methanol extracts of *C. odorata* and *T. ciliata*

**FIGURE 1:**
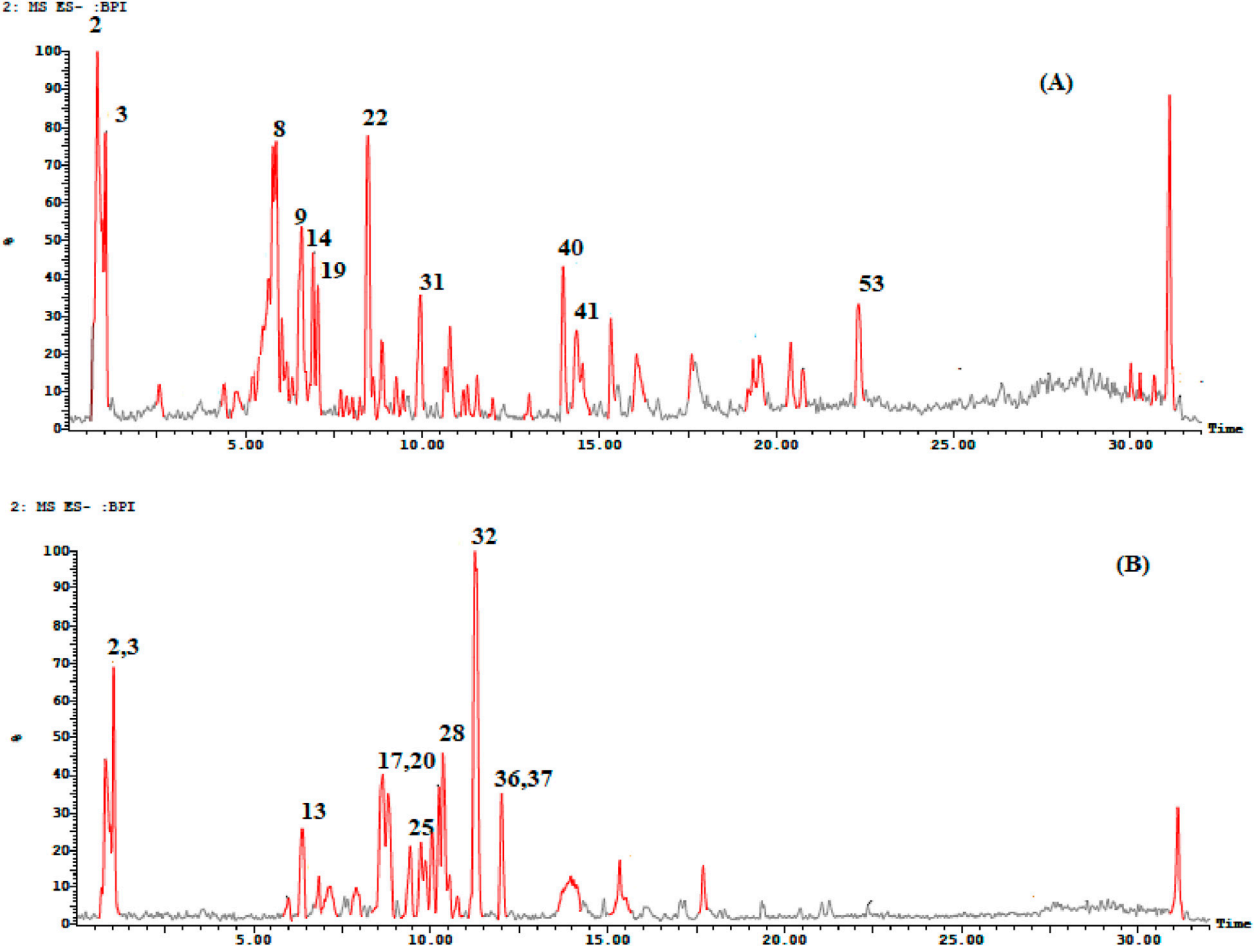
TIC of the 80% methanol extract of **(A)** *C. toona* and **(B)** *C. odorata*.

The search for natural therapeutic agents is increasing nowadays due to their advantages of being safe, effective, and readily available. ThegGenus *Cedrela* is one of the well-known and understudied genera with many beneficial health activities, including cytotoxic (Choi et al., 2015), antiviral, hepatoprotective (leaves) (Asaad et al., 2021), anti-microbial (leaves) (Paritala et al., 2015), hypoglycemic (bark) (Giordani et al., 2015), antioxidant (bark) (Shah and Patel, 2021; Kumari and Kakkar, 2008), anti-leishmania (Takahashi et al., 2004), and anti-larval activities (Koul, 1983). The UPLC-ESI/MS^n^ technique was used to identify the phytoconstituents of the 80% methanol extracts of *Cedrela* species (*Cedrela odorata* and *Toona ciliata*). As shown in Figure 1 and Table 1, the UPLC-ESI/MS^n^ analysis resulted in the tentative identification of 55 compounds with their specific fragmentation patterns (% identification ranged from 88.61% to 89.38%). Different phytochemical classes were detected, including flavonoids, phenylpropanoids, terpenes, and tannins. The tentatively identified compounds can be summarized as follows. The fragmentation patterns of the identified compounds are illustrated in Supplementary Figures S1–S14.

**TABLE 1:** UPLC-ESI/MS^n^ metabolic profiling of the phytoconstituents of *T*. *ciliata* and *C. odorata* in the negative and positive ion modes.

No.	Compound	Molecular formula	Class	R_t_ (min.)	[M-H]^-^(*m/z*)	[M + H]^+^/[M + H + Na]^+^ (*m/z*)	MS/MS fragments	% composition		Ref.
								TC	CO	
1	Afzelechin	C_15_H_14_O_5_	Flavonoid	0.70	273	—	—	—	1.81	Zaghloul et al. (2023)
2	Fragment	—	—	0.71	377	381	—	**14.76**	**8.79**	Benayad et al. (2014)
3	Icariside I	C_27_H_30_O_11_	Flavonoid	1.05	531	—	300, 388, 219, and 101	**4.33**	**8.00**	Ren and Long (2017)
4	Chlorogenic acid derivative	—	Phenylpropanoid	2.57	451	—	298, 276, 191, 169, 108, and 71	0.51	—	Simirgiotis et al. (2015)
5	Kaempferol-*O*-pentoside	C_20_H_18_O_10_	Flavonoid	4.39	431	433	285	**2.87**	**3.52**	Chen H. J et al. (2011)
6	Acacetin pento-hexoside	C_28_H_32_O_14_	Flavonoid	4.77	591	—	289, 265, 255, 119, 133, and 103	0.87	—	Al-Yousef et al. (2020)
7	Fragment of (epi)gallocatechin	—	Tannin	5.21	467	—	409, 347, 289, 283, 255, 101, and 99	0.58	—	Escobar-Avello et al. (2019)
8	Fragment of ursolic acid	—	Triterpene	5.40	411	—	265, 247, 179, 163, and 119	**11.03**	—	Chen H. J et al. (2011)
9	Apigenin derivative	—	Flavonoid	5.87	521	—	285, 236, 196, 183, and 161	**9.60**	—	Rini Vijayan and Raghu (2019)
10	Rutin	C_27_H_30_O_16_	Flavonoid	5.99	609	—	315, 301, 209, 188, and 83	—	1.97	Simirgiotis et al. (2015)
11	Quercetin-*O*-hexoside	C_21_H_20_O_12_	Flavonoid	6.16	463	—	357, 310, 301, 308, 271, and 255	0.76	—	Simirgiotis et al. (2015)
12	Salvianolic acid A	C_26_H_22_O_10_	Miscellaneous	6.32	493	—	403, 165, 133, 121, 101, and 99	0.25	—	Barros et al. (2013)
13	Kaempferol-deoxyhexosyl-hexoside	C_27_H_29_O_14_	Flavonoid	6.38	593	—	285, 227, 209, and 169	—	**5.12**	Elhawary et al. (2021)
14	Quercetin-3-*O*-pentoside	C_20_H_18_O_11_	Flavonoid	6.59	447	449	372, 153, 301, 284, 271, 254, and 239	**5.80**	—	Cunja et al. (2014), Sobeh et al. (2016)
15	Aloeresin B	C_19_H_22_O_9_	Anthraquinone	6.70	393	—	307, 277, 163, and 113	0.37	—	El Sayed et al. (2016b)
16	Quinic acid derivative	—	Phenylpropanoid	6.81	441	—	265, 235, 191, 175, and 89	0.36	2.12	Chen Q et al. (2011)
17	Quercetin-*O*-acetyl-hexoside	C_23_H_22_O_13_	Flavonoid	7.17	505	—	345, 301, 293, 239, 161, and 103	—	**3.00**	Elhawary et al. (2021)
18	3,5-di-*O*-Caffeoylquinic acid	C_25_H_24_O_12_	Phenylpropanoid	7.69	515	—	321, 303, 271, 261, and 191	2.14	—	Chen Q et al. (2011)
19	Chicoric acid derivative	—	Phenylpropanoid	7.86	473	—	328, 266, 243, 209, and 101	**3.33**	—	Chen H. J et al. (2011)
20	Caffeic acid hexoside derivative	—	Phenylpropanoid	7.90	533	—	388, 371, 330, 319, 299, and 269	—	**7.36**	Elhawary et al. (2021)
21	3-Methyl-epigallocatechin gallate	C_23_H_20_O_11_	Tannin	8.23	471	—	289 and 140	0.23	—	Bastos et al. (2007)
22	Kaempferol acetyl-hexoside	C_23_H_22_O_12_	Flavonoid	8.46	489	—	337 and 285	**7.09**	—	Kramberger et al. (2020)
23	Chrysoeriol-*O*-hexouronic acid	C_22_H_20_O_12_	Flavonoid	8.61	475	—	—	0.73	—	El Sayed et al. (2016a)
24	(epi)afzelechin–(epi)catechin dimer	C_30_H_25_O_11_	Tannin	9.25	561	—	273	1.02	—	Gu et al. (2003)
25	Manniflavanone	C_30_H_22_O_13_	Flavonoid	9.43	589	—	443, 399, 341, 331, 306, 287, 265, 123, and 113	—	**7.06**	Reed (2009)
26	Fragment of caffeoyl diferuloylquinic acid	—	Phenylpropanoid	9.56	—	545	—	0.53	—	Schmeda-Hirschmann et al. (2015)
27	13-*O*-Phenylacetyl-12- deoxyphorbol-20-acetate	C_30_H_36_O_7_	Miscellaneous	10.25	531	—	265, 119, and 101	—	**5.20**	Ghani and Badr (2020)
28	Ganolucidic acid B	C_30_H_46_O_6_	Triterpene	10.37	501	503	213	—	**7.24**	Yang et al. (2007)
29	(epi)Catechin-ethyl dimer	—	Tannin	10.64	605	—	289	1.14	—	Rockenbach et al. (2012)
30	Apigenin 6-*C*-pentoside-8-*C*-pentoside	C_25_H_26_O_13_	Flavonoid	10.76	547	—	456, 425, 417, 285, 263, 237, and 135	—	2.03	Marzouk et al. (2019)
31	Pallidol	C_28_H_22_O_6_	Stilbene dimer	10.78	453	—	364, 245, 240, and 111	2.64	—	Escobar-Avello et al. (2019)
32	Ferulic acid derivative	—	Phenylpropanoid	11.28	517	551	266, 255, 241, and 212	0.53	**13.79**	Bystrom et al. (2008)
33	Abscisic acid-*O*-hexoside-HMG	—	Tannin	11.54	585	—	—	0.76	—	El-Sayed et al. (2017)
34	7,8-Dihydro-3- oxo-*α*-ionol *β*-D-hexoside	C_13_H_22_O_2_	Miscellaneous	11.88	—	373	—	—	1.11	El-sayed et al. (2021)
35	Arbutin	C_12_H_16_O_7_	Flavonoid	11.98	311	—	—	0.36	—	Jia et al. (2017)
36	A-type proanthocyanidin dimer	C_30_H_24_O_12_	Tannin	12.00	575	—	459, 443, 211, and 175	—	**5.65**	Reed (2009)
37	Secoisolariciresinol guaiacylglyceryl ether	C_30_H_38_O_10_	Sesquilignan	12.25	—	559	403, 379, and 337	—	**4.38**	Patyra et al. (2022)
38	Procyanidin dimer	C_30_H_26_O_12_	Tannin	13.01	573	—	—	0.51	—	Reed (2009)
39	Methyl trigalloyl hexose	C_28_H_27_O_18_	Tannin	13.96	649	—	539, 529, 499, 455, and 359	—	**5.95**	El-sayed et al. (2021)
40	Eicosanoyl derivative of 12-ursen-3-ol	C_50_H_88_O_2_	Triterpene	13.97	721	—	577, 411, and 289	2.79	—	Xiao et al. (2023)
41	Fragment of arbutin	—	Flavonoid	14.35	293	—	163, 113, and 89	2.33	—	Jia et al. (2017)
42	Heliarzanol 1	C_24_H_30_O_8_	Pyrone derivative	16.06	445	—	206, 193, 164, and 112	2.22	—	Kramberger et al. (2020)
43	Apigenin 6-*C*-*α*-pentoside-8-*C*-*β*-hexoside (isoviolanthin)	C_27_H_30_O_14_	Flavonoid	16.52	—	579	374, 259, and 199	1.42	1.15	Marzouk et al. (2019)
44	Chlorogenic acid	C_16_H_18_O_9_	Phenylpropanoid	17.91	—	353	299, 251, and 209	0.47	1.38	Elhawary et al. (2021)
45	Fragment of rutin	—	Flavonoid	19.20	423	—	379, 327, and 294	**3.16**	—	Shrestha et al. (2017)
46	Eriodictyol-7-*O*-hexoside	C_21_H_22_O_11_	Flavonoid	19.38	—	451	264, 203, and 169	—	0.78	Ashraf et al. (2020)
47	Caffeoyl-2-hydroxyethane-1,1,2-tricarboxylic acid	—	Phenylpropanoid	20.40	339	—	265, 179, and 103	1.68	—	Ye et al. (2005), Ben Said et al. (2017)
48	Fragment of chlorogenic acid	—	Phenylpropanoid	20.73	313	—	—	0.98	—	Ibrahim et al. (2015)
49	Acetyl-*O*-galloyl hexose	C_14_H_19_O_10_	Tannin	20.82	—	375	—	0.60	—	El-sayed et al. (2021)
50	Rhamnocitrin-*O*-rutinoside	—	Flavonoid	20.96	—	609	315, 209, and 188	1.94	—	ElKhateeb et al. (2019)
51	Trigalloyllevohexosan	—	Tannin	21.13	—	621	509, 445, and 223	—	0.62	El-sayed et al. (2021)
52	Myricetin-*O*-hexosyl-*O*-hexouronoside	—	Flavonoid	22.32	655	—	285 and 179	**3.15**	—	Affes et al. (2021)
53	Isorhamnetin 7-*O*-[3-hydroxy-3- methylglutaroyl]-hexoside	—	Flavonoid	22.87	—	623	315, 270, 168, and 75	**11.07**	**6.57**	López-Angulo et al. (2018)
54	Galloyl ester of 5,6,7- trihydroxy-2,3- dihydrocyclopenta [b]chrom ene-1,9-dione-3-carboxylic acid hexoside	—	Miscellaneous	24.07	—	607	547 and 460	**20.98**	**17.28**	Fraternale et al. (2015)
55	12-*O*-*β*-D-hexoside deriv. of 8,11,13-Abietatriene-3,11,12,16-tetrol	C_26_H_40_O_9_	Diterpene	30.01	597	—	—	0.50	—	Lee et al. (2016)
	**% Identification** **ESI −ve mode** **ESI + ve mode**							**89.38** **37.00**	**88.61** **33.27**	

Bold font indicates high percentage of compounds.

#### 3.1.1 Flavonoids

**FIGURE 2:**
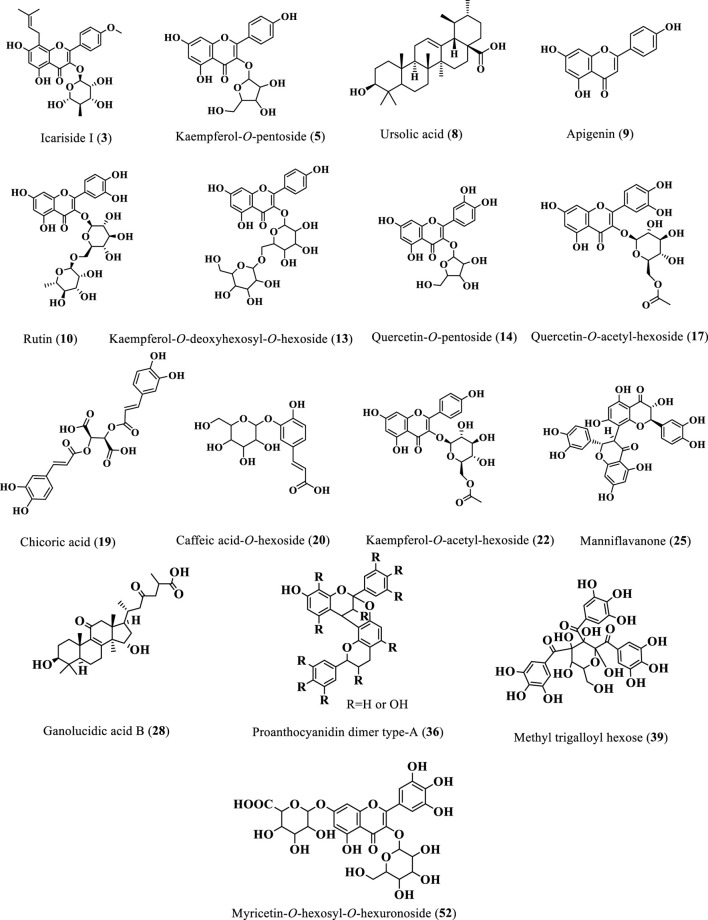
Structures of the major identified phytoconstituents of the 80% methanol extracts of *T*. *ciliata* and *C. odorata*.

Twenty-two flavonoids were tentatively identified from the extracts of *Cedrela odorata* and *Toona ciliata* representing the major class of identified compounds (Table 1; Figure 2). A deprotonated molecular ion peak was shown at [M-H]^−^ *m/z* 273 (R_t_ = 0.70 min, only in *C. odorata*) and was tentatively identified as afzelechin (Zaghloul et al., 2023). Another flavonoid peak was traced in the ESI negative ion mode at *m/z* 531 (R_t_ = 1.05 min, 4.33% *T. ciliata,* 8.00% *C. odorata*) with fragments at *m/z* 300, 388, 219, and 101 thus it was assigned to icariside I (Ren and Long, 2017). In addition to that, 16 different flavonoid glycosides were tentatively identified at different retention times and can be detailed as follows. A molecular ion peak was detected at [M-H]^−^ *m/z* 431 and [M + H]^+^ *m/z* 433 with one major fragment at *m/z* 285 due to loss of kaempferol aglycone and was tentatively identified as kaempferol-3-*O*-pentoside (Chen Q. et al., 2011). Another pentoside was detected at *m/z* 447 in the ESI negative ion mode and *m/z* 449 in the ESI positive ion mode with daughter peaks at m/z 372, 153, 301, 284, 271, 254, and 239, where the quercetin aglycone was clear at *m/z* 301; thus, it was defined as quercetin-3-*O*-pentoside (5.80% *T*. *ciliata*) (Cunja et al., 2014; Sobeh et al., 2016). Two acetyl glycosides were detected at *m/z* 505 and *m/z* 489 and were tentatively assigned to quercetin-*O*-acetyl-hexoside (MS/MS at *m/z* 345, 301, 293, 239, 161, and 103; 3.00%, *C. odorata*) and kaempferol acetyl-hexoside (MS/MS at *m/z* 337 and 285; 7.09%, *T*. *ciliata*), respectively (Elhawary et al., 2021; Kramberger et al., 2020).

A deprotonated peak was shown at *m/z* 463 in the ESI negative mode with fragments at *m/z* 357, 310, 301, 308, 271, and 255 and was found to be a quercetin derivative, namely, quercetin-*O*-hexoside (Simirgiotis et al., 2015). Similarly, another kaempferol derivative showed a molecular ion peak at [M-H]^−^ *m/z* 593 (5.12%, *C. odorata*) and was tentatively defined as kaempferol-deoxyhexosyl-hexoside (Elhawary et al., 2021). Compound 10 showed a deprotonated peak at *m/z* 609 in the ESI negative mode and was tentatively identified as rutin (Simirgiotis et al., 2015); it further showed one of its fragments at *m/z* 423 (Shrestha et al., 2017). In the same context, a peak was detected at [M-H]^−^ *m/z* 311 for the glycoside arbutin (Jia et al., 2017), together with its fragment at *m/z* 293 (Jia et al., 2017). Three compounds belonging to the flavonoid apigenin were detected at *m/z* 521 (9.60% *T*. *ciliata*) (Rini Vijayan and Raghu, 2019) and m/z 547 (Marzouk et al., 2019), both in the ESI negative ion mode for an apigenin derivative and apigenin 6-*C*-pentoside-8-*C*-pentoside, respectively, and their identity was confirmed by their fragmentation patterns detected as (*m/z* 285, 236, 196, 183, and 161) and *(m/z* 456, 425, 417, 285, 263, 237, and 135), respectively, where the kaempferol aglycone fragment was traced at *m/z* 285 in the two compounds. In addition, apigenin 6-*C*-*α*-pentoside-8-*C*-*β*-hexoside (isoviolanthin) was traced in the ESI positive ion mode at *m/z* 579 (Marzouk et al., 2019). Compound 6 presented a deprotonated peak at *m/z* 591 and MS/MS at *m/z* 289, 265, 255, 119, 133, and 103 and was tentatively assigned to the glycoside acacetin pento-hexoside (Al-Yousef et al., 2020).

Moreover, chrysoeriol-7-*O*-hexouronic acid showed its deprotonated peak at *m/z* 475 (El Sayed et al., 2016a). Compound 25 was tentatively defined as manniflavanone with a deprotonated peak at *m/z* 589 in the ESI negative ion mode and daughter peaks at *m/z* 443, 399, 341, 331, 306, 287, 265, 123, and 113 (7.06%, *C. odorata*) (Reed, 2009). Only one myricetin derivative was tentatively detected at [M-H]^−^ *m/z* 655 and was identified as myricetin-*O*-hexosyl-*O*-hexouronoside (MS/MS at *m/z* 285 and 179; 3.15%, *T*. *ciliata*) (Affes et al., 2021). Three different flavonoid glycosides were tentatively traced in the ESI positive ion mode at *m/z* 451, *m/z* 609, and *m/z* 623 and were assigned to eriodictyol-7-*O*-hexoside (Ashraf et al., 2020), rhamnocitrin-*O*-rutinoside (ElKhateeb et al., 2019), and isorhamnetin 7-*O*-[3-hydroxy-3- methylglutaroyl]-hexoside (López-Angulo et al., 2018), respectively.

#### 3.1.2 Tannins

As shown in Table 1 and Figure 2, ten tannins and their fragments were identified from the two *Cedrela* extracts. A deprotonated peak was detected at *m/z* 471 in the ESI negative mode and was attributed to the compound, 3-methyl-epigallocatechin gallate (Bastos et al., 2007). Moreover, a fragment of (epi) gallocatechin was traced at *m/z* 467 (Escobar-Avello et al., 2019). In addition to that, two catechin-containing dimers were tentatively identified as compounds 24 and 29. The former showed its peak at [M-H]^−^ *m/z* 561 and was assigned to (epi) afzelechin–(epi) catechin dimer (Gu et al., 2003), while the latter had a peak at [M-H]^−^ *m/z* 605 and was defined to be (epi) catechin-ethyl dimer (Rockenbach et al., 2012).

Compound 33 with a deprotonated peak at the *m/z* 585 in negative mode was identified as abscisic acid-*O*-hexoside-HMG (El-Sayed et al., 2017). On the other hand, compounds 36 and 38 showed deprotonated peaks at *m/z* 575 (5.65% *C. odorata*) and *m/z* 573 in the ESI negative mode (MS/MS at *m/z* 459, 443, 211 and 175) and were identified as A-type proanthocyanidin dimer and procyanidin dimer, respectively (Reed, 2009). Regarding the hydrolysable tannins, three of them were traced at *m/z* 649 (ESI negative), m/z 375 (ESI positive), and m/z 621 (ESI positive) and were tentatively assigned to methyl trigalloyl hexose (5.95% *C. odorata*), acetyl-*O*-galloyl hexose, and trigalloyl hexose, respectively (El-sayed et al., 2021).

#### 3.1.3 Phenylpropanoids

Ten phenylpropanoids were traced (Table 1; Figure 2) and can be detailed here; compound 16 showed a deprotonated peak at [M-H]^−^ *m/z* 441 and was identified as a quinic acid derivative (Chen H. J. et al., 2011). Similarly, compound 18 was also a quinic acid-containing phenylpropanoid with a parent peak at *m/z* 515 in the ESI negative mode and was defined as 3,5-di-*O*-caffeoylquinic acid (Chen H. J. et al., 2011). A fragment of caffeoyl diferuloylquinic acid was detected at [M + H]^+^ *m/z* 545 (Schmeda-Hirschmann et al., 2015). Moreover, another parent peak was traced at *m/z* 533 with its fragments at *m/z* 388, 371, 330, 319, 299, and 269 (ESI negative, 7.36% *C. odorata*) and was attributed to the presence of a caffeic acid hexoside derivative (Elhawary et al., 2021). Compounds 4,44 and 48 were showing parent peaks at *m/z* 451 (ESI–ve), *m/z* 353 (ESI + ve), and *m/z* 313 (ESI–ve) and were tentatively identified as a chlorogenic acid derivative (Simirgiotis et al., 2015), chlorogenic acid (Elhawary et al., 2021), and a fragment of chlorogenic acid (Ibrahim et al., 2015), respectively. The aforementioned compounds shared the presence of one characteristic fragment at *m/z* 191 due to the quinic acid fragment. A deprotonated molecular ion peak was detected at [M-H]^−^ *m/z* 473 with MS/MS at *m/z* 328, 266, 243, 209, and 101 (3.33% *T*. *ciliata*) and was identified as a chicoric acid derivative (Chen H. J. et al., 2011). Compound 32 showed a parent peak at [M-H]^−^ *m/z* 517 and [M + H + CH_3_O]^+^ *m/z* 551 (MS/MS at *m/z* 266, 255, 241, and 212) and was assigned to a ferulic acid derivative (13.79% *C. odorata*) (Bystrom et al., 2008). Another phenylpropanoid peak was detected at the *m/z* 339 in ESI negative mode and was tentatively found to be caffeoyl-2-hydroxyethane-1,1,2-tricarboxylic acid (Ye et al., 2005; Ben Said et al., 2017).

#### 3.1.4 Triterpenes

Two triterpenes were detected (Table 1; Figure 2). The first one presented a parent peak at *m/z* 501 in the ESI negative mode and *m/z* 503 in the ESI positive mode with one fragment at *m/z* 213 (7.24% *C. odorata*), and it was defined as the triterpene, ganolucidic acid B (Yang et al., 2007). The second triterpene peak was shown at *m/z* 721 in the ESI negative mode and was tentatively assigned to the eicosanoyl derivative of 12-ursen-3-ol (Xiao et al., 2023).

#### 3.1.5 Diterpenes

Only one diterpene derivative was traced from the *T*. *ciliata* extract (Table 1; Figure 2). This diterpene was identified as 12-*O*-*β*-D-hexoside derivative of 8,11,13-abietatriene-3,11,12,16-tetrol, and it showed a deprotonated peak at [M-H]^−^ *m/z* 597 (Lee et al., 2016).

Herein, 55 phytoconstituents were tentatively identified and quantified from two species of *Cedrela,* namely, *C. odorata* and *T*. *ciliata*. This study represents the first study with in-depth phytochemical profiling of two understudied *Cedrela* species through UPLC/MS analysis in both positive and negative ion modes, accompanied with identification and quantification of their metabolites with fragmentation patterns and comparison to the available literature.

Upon reviewing the LC/MS literature on genus *Cedrela*, few reports were found on these two species in particular. Bark and heartwood extracts of *T*. *ciliata* showed the presence of toonacillin, 6-hydroxy-toonacillin, and geranyl geraniol as its fatty esters (Shah and Patel, 2021). Limonoids (triterpenes), proanthocyanidins, flavonoids, and phenols were evaluated from 70 samples of *C. odorata* leaves through HPLC-UV-DAD, and it was found that the main identified components were kaempferol glycoside and catechin, while *β*-elemene, E-caryophyllene, aromadendrene, *α*-humulene, *γ*-cadinene, D-germacrene, bicyclogermacrene, *α*-tocopherol, and *β*-sitosterol were the main detected compounds using GC/MS analysis for the same samples (Bellone et al., 2021). In addition to that, other relevant members of the family Meliaceae were evaluated using LCMS analysis, and a study on *Melia azedarach* L. was performed utilizing UPLC/MS/MS analysis where 29 components were identified, including flavonoid *O*-glycosides, simple flavonoids, triterpenoid saponins, and cardenolides as the major constituents (Saeed et al., 2022). Moreover, *Azadirachta indica* (the neem plant) leaf extract was analyzed through UPLC/MS where limonoids were revealed as the major contributing components of its metabolic profile (Rangiah et al., 2016).

Four triterpenes were isolated from *C. odorata* wood ethanol extract including gedunin, 3*β*-O-*β*-D-glucopyranosyl-24-methyllenecholesterol, oleanolic acid, sitosterol, n-octacosanol, and threo-23,24,25-trihydroxytirucall-7-en-3-one (Campos et al., 1991). In addition to that, calamenene, cycloeucalenol, sitosterol, stigmasterol, campesterol, gedunin, 7-deacetylgedunin, 7-deacetoxy-7-oxogedunin, methylangolensate, febrifugin, azadiradione, 20,21,22,23-tetrahydro-23-oxoazadirone, 3*β*-deacetylfissinolide and catechin, 1 *α*-methoxy-1,2-dihydrogedunin, and 3*β*-*O*-*β*-D-glucopyranosylcycloeucalenol were isolated from the stem extract of *C. odorata* (de Paula et al., 1997).

7-deoxo-7*α*,11*β*-diacetoxykihadanin A; 1,2-dihydro-7-deoxo-1*α*,7*α*,11*β*-triacetoxykihadanin A; cedrelosin F, 11*β*-acetoxylimonol; and 11*β*-acetoxycedrelosin B were defined from the stem bark extract of *C. odorata* (Bellone et al., 2021). Similarly, 4,5-dihydroblumenol A, 7-megastigmene-3α,6,9-triol, catechin, scopoletin, homovanillic alcohol, and 2-(3,4-dimethoxyphenyl)-ethyl-*O*-*β*-D-glucopyranoside were isolated from the stem bark of *C. odorata* (Muñoz Camero et al., 2018). Through HPLC, cedrodorin, 6-acetoxycedrodorin, 6-deoxy-9*α*-hydroxycedrodorin, and 9*α*-hydroxycedrodorin were isolated from the leaves of *C. odorata* (Veitch et al., 1999). Moreover, methylangolensate was detected from the heartwood of *C. odorata* (Chan et al., 1967). From the extracts of the leaves and twigs of *C. odorata*, cedrodorols A and B were detected (Wu et al., 2014). A triterpenoid, belonging to the limonoids, called odoratin was isolated from *C. odorata* (Chan et al., 1966).

### 3.2 Assessment of *in vitro* anti-diabetic activity

**FIGURE 3:**
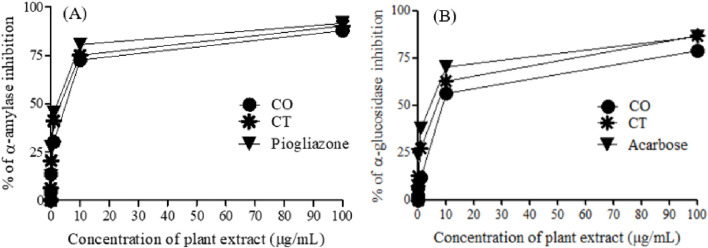
*α*-Amylase **(A)** and *α*-glucosidase **(B)** inhibition of 80% methanol leaf extracts of *C. odorata* and *T*. *ciliata*, compared to standard drugs.

One of the therapeutic strategies for DM management is to retard hyperglycemia post-ingestion which can be achieved by inhibiting enzymes relevant to carbohydrate digestion like *α*-amylase and *α*-glucosidase (Ali et al., 2006). Thus, *α*-amylase and *α*-glycosidase enzyme inhibition assays were used to test *in vitro* anti-diabetic activities of the leaf extracts of *C. odorata* and *T*. *ciliata* (0.01, 0.10, 1.10, and 100 μg/mL), as shown in Table 2 and Figure 3.

**TABLE 2:** *α*-Amylase and *α*-glucosidase inhibitory activities of 80% methanol leaf extracts of *C. odorata* and *T*. *ciliata*, compared to standard drugs.

Concentration (µg/mL)	*α*-Amylase inhibition (%)			*α*-Glucosidase inhibition (%)		
	CO	CT	Pioglitazone	CO	CT	Acarbose
0.01	4.45 ± 1.56	6.29 ± 1.60	12.50 ± 2.05	2.08 ± 2.63	7.46 ± 1.70	7.41 ± 1.23
0.10	13.50 ± 2.60	20.50 ± 2.35	27.70 ± 1.28	6.44 ± 1.95	12.80 ± 2.02	23.60 ± 2.01
1.00	30.20 ± 1.29	41.20 ± 1.73	45.40 ± 2.45	11.80 ± 1.29	27.40 ± 1.63	37.60 ± 0.42
10.00	72.60 ± 2.22	75.10 ± 1.96	80.60 ± 0.23	56.30 ± 2.56	62.90 ± 0.58	70.20 ± 1.28
100.00	87.90 ± 1.67	90.30 ± 2.08	91.70 ± 1.05	78.60 ± 1.89	86.60 ± 1.87	86.10 ± 0.75
IC_50_ (µg/mL)	4.83 ± 0.01	3.50 ± 0.03	2.17 ± 0.23	7.17 ± 0.01	6.50 ± 0.69	4.83 ± 1.02

*Values represent means ± SD (standard deviations) for triplicate experiments.

The tested extracts and standard anti-diabetic drugs (pioglitazone and acarbose) showed concentration-dependent inhibition of *α*-amylase and *α*-glucosidase enzymatic activities. The leaf extracts of *C. odorata* and *T*. *ciliata* markedly inhibited *α*-amylase activities with IC_50_ values of 4.83 ± 0.01 and 3.50 ± 0.03 μg/mL, respectively, compared to standard pioglitazone (standard *α*-amylase inhibitor; 2.17 ± 0.23 μg/mL). Furthermore, they showed significant *α*-glycosidase inhibitory properties with IC_50_ values of 7.17 ± 0.01 and 6.50 ± 0.69 μg/mL, respectively, compared to acarbose (*α*-glycosidase inhibitor; IC_50_ = 4.83 ± 1.02 μg/mL). In this study, the leaf extract of *T*. *ciliata* demonstrated larger inhibition values of 90.30% ± 2.08% and 86.60% ± 1.87% for *α*-amylase and *α*-glucosidase, respectively, than those of *C. odorata* (87.90% ± 1.67% and 78.60% ± 1.89%) at concentration of 100 μg/mL. The results suggest the anti-diabetic potential of the tested extracts. Based on inhibiting the activities of these enzymes, they could delay carbohydrate digestion and prolong the overall time for carbohydrate digestion, resulting in a reduction in the rate of glucose absorption and consequently blunting the postprandial blood glucose rises, i.e., making food have lower glycemic index (Ahmad et al., 2012). Furthermore, the extract of *C. odorata* showed superior activity than that of *T*. *ciliata*; it may be attributed to the high percentage of flavonoids in *C. odorata* which acts synergistically to inhibit the activities of these metabolizing enzymes.

With the same line of our study, previous research reported that the hydroethanolic extract of *C. odorata* could blunt the postprandial glycemic surge in the streptozotocin-induced diabetic rat model (Giordani et al., 2015). According to our results, the reported anti-hyperglycemic activity was attributed to the inhibition of *α*-amylase and *α*-glucosidase activities. Thus, the anti-diabetic efficacy of Cedrela extracts could be due to the existence of active constituents with diverse mode of actions at the molecular level, comprising *α*-amylase and *α*-glycosidases.

Ursolic acid (**8**) as one of the major identified compounds (Figure 2) displays a positive effect on reducing blood glucose levels and alleviating the diabetes-related complications in several diabetic animal models such as streptozotocin-nicotinamide-induced diabetic mice (Lee et al., 2010), streptozotocin-induced diabetic mice fed a high-fat diet (Jang et al., 2009), and streptozotocin-induced diabetic mice (Jang et al., 2010). In the same manner, icariside I (**3**), was previously obtained from *Herba Epimedii* (Berberidaceae) and enhanced the type-2 diabetes mellitus profile in db/db mice in a dose-dependent manner (Li et al., 2022). Furthermore, the leaf extract of *Simarouba glauca* (Simaroubaceae) was reported to be rich in kaempferol-*O*-pentoside (**5**) and effectively inhibited *α*-glucosidase activities with IC_50_ value of 2.4 0.4 μg/mL (Mugaranja and Kulal, 2020). Rutin (**10**) was found to reduce the carbohydrate absorption from the small intestine and inhibit tissue gluconeogenesis, along with glucose uptake activation (Ghorbani, 2017). Furthermore, it was reported to enhance insulin release from beta cells and protect Langerhans islet from degeneration via antioxidant activity (Jadhav and Puchchakayala, 2012).

The quercetin aglycone was predominately identified in some compounds like **11**, **14**, and **17,** as shown in Table 1. A certain study reported that quercetin markedly inhibited α-amylase and α-glucosidase activities (Shen et al., 2023). Moreover, a combination of quercetin and rutin (**10**) displayed higher synergistic inhibition against these enzymes than the individual compounds (Oboh et al., 2015).

A combination of kaempferol (found in **5**, **13** and **22**) and myricetin (found in **52**) showed synergistic anti-diabetic activity in STZ-activated diabetes in rats via antioxidant and anti-inflammatory activities (Al-Abbasi and Kazmi, 2023).

Caffeic acid (found in **18**, **20**, **26,** and **47**) was reported to exhibit anti-hyperglycemic properties in C57BL/KsJ-*db/db* mice via enhanced glucokinase activity and glycogen content and concurrently depressed glucose-6-phosphatase and phosphoenolpyruvate carboxykinase activities accompanied by decreased glucose transporter-2 expression in the liver (Jung et al., 2006).

### 3.3 Molecular docking studies

#### 3.3.1 Determination of affinity scores of the identified major components with *α*-amylase and *α*-glucosidase

Molecular docking, a computational technique, is utilized to position computer-generated 3D structures of small ligands within a receptor structure in various orientations, conformations, and positions. These ligands interact with the receptor through binding energy, providing insights into their pharmacological actions. In our docking simulation, the test ligands myricetin-*O*-hexosyl-*O*-hexuronoside, rutin, and ursolic acid exhibited the top three higher binding affinities with docking scores of −9.1, −9.3, and −9.4 kcal/mol, respectively, toward the *alpha*-amylase enzyme. Conversely, the standard ligand pioglitazone showed a binding affinity of −7.9 kcal/mol with the *alpha*-amylase enzyme. On the other hand, the test ligands methyl trigalloyl hexose, myricetin-*O*-hexosyl-*O*-hexuronoside, and rutin revealed the top three higher binding affinities with docking scores of −9.5, −9.4, and −9.6 kcal/mol, respectively, toward the *alpha*-glucosidase enzyme. In contrast, the standard ligand acarbose showed a binding affinity of −7.6 kcal/mol with the *alpha*-glucosidase enzyme. The binding affinities of other ligands with *alpha*-amylase and *alpha*-glucosidase enzymes are shown in Table 3.

**TABLE 3:** Molecular docking affinity scores among the selected ligands with *α*-amylase and *α*-glucosidase enzymes.

Ligand	Molecular docking affinity (kcal/mol)	
	*α*-Amylase (4GQR)	*α*-Glucosidase (3TOP)
Apigenin	−8.2	−9.1
Caffeic acid hexoside	−7.9	−7.5
Chicoric acid	−8.6	−8.8
Ganolucidic acid B	−8.4	−8.4
Icariside I	−7.9	−8.4
Kaempferol acetyl-hexoside	−7.7	−9.1
Kaempferol-3-*O*-pentoside	−7.6	−9.2
Kaempferol-deoxyhexosyl-hexoside	−9.0	−8.7
Manniflavanone	−8.8	−9.2
Methyl trigalloyl hexose	−8.8	−9.5
Myricetin-*O*-hexosyl-O-hexuronoside	−9.1	−9.4
Quercetin-3-*O*-pentoside	−7.8	−8.8
Quercetin-*O*-acetyl-hexoside	−8.1	−9.3
Rutin	−9.3	−9.6
Ursolic acid	−9.4	−8.6
Acarbose	-	−7.6
Pioglitazone	−7.9	-

#### 3.3.2 Prediction of the active site of enzyme–ligand interactions

##### 3.3.2.1 Top three test ligands and pioglitazone with α-amylase enzyme interactions

**FIGURE 4:**
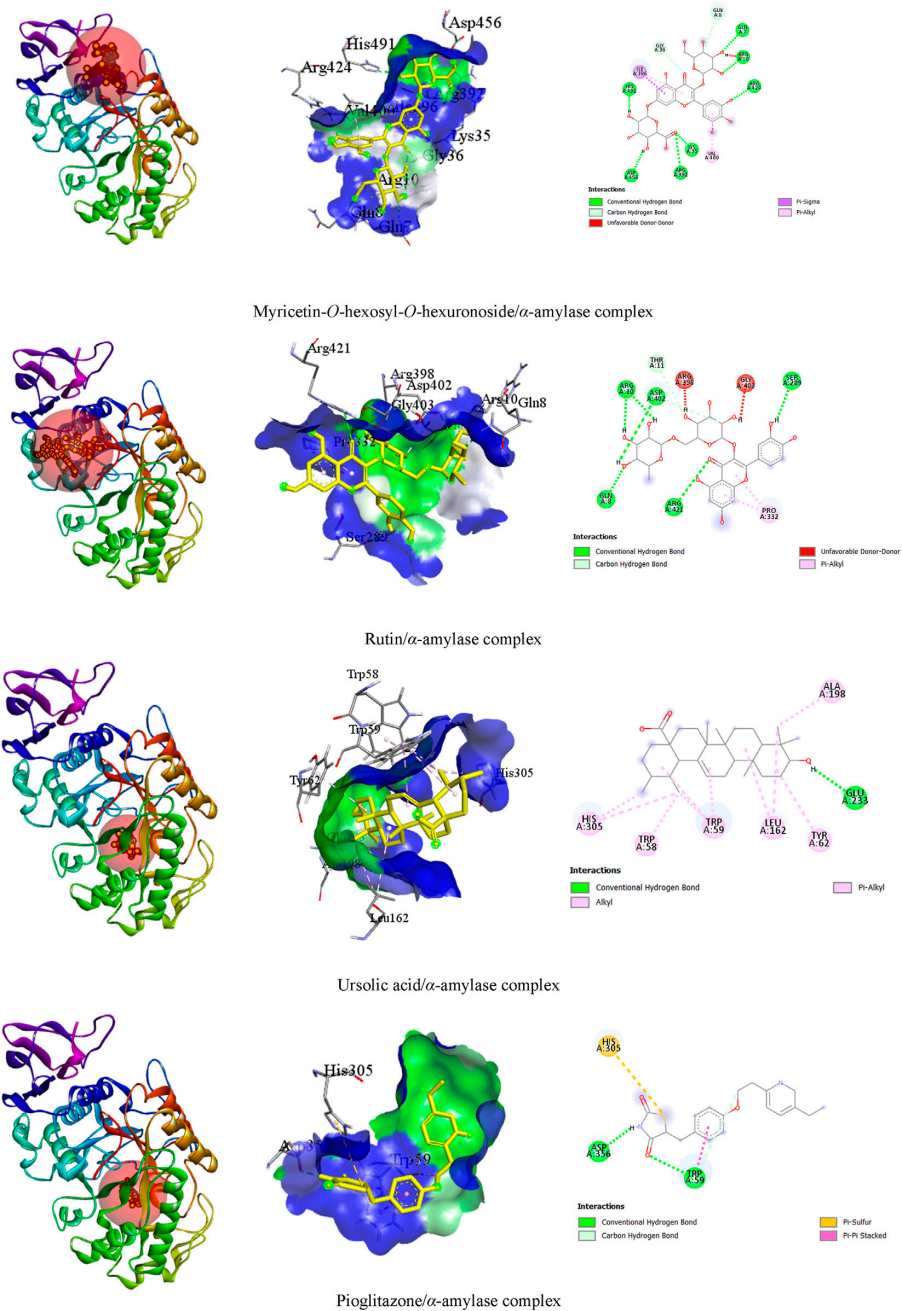
The 3D and 2D views of the top three ligand interactions with the *α*-amylase enzyme in the binding site highlight non-bond interactions.

In our visualization of enzyme–ligand interactions, we observed that the test ligands myricetin-*O*-hexosyl-*O*-hexuronoside, rutin, and ursolic acid formed numerous hydrogen bonds and other bonds with amino acids in the *α*-amylase enzyme’s binding pocket. Specifically, myricetin-*O*-hexosyl-*O*-hexuronoside created nine hydrogen bonds with HIS A:491, ASP A:456, GLN A:7, ARG A:10, LYS A:35, ARG A:392, ARG A:424, GLN A:8, and GLY A:36 amino acids, along with forming other bonds with ILE A:396 and VAL A:400 amino acids. Similarly, rutin formed six hydrogen bonds with ASP A:402, ARG A:10, SER A:289, GLN A:8, ARG A:421, and THR A:11 amino acids. Additionally, rutin established a hydrophobic bond with the PRO A:332 amino acid in the enzyme’s binding site. Furthermore, ursolic acid showed one hydrogen bond with GLU A:233 amino acid and exhibited several hydrophobic bonds with LEU A:162, ALA A:198, TRP A:58, TRP A:59, and TYR A:62, as well as HIS A:305 amino acid. In contrast, pioglitazone interacted with the *alpha*-amylase enzyme by forming two hydrogen bonds with ASP A:356 and TRP A:59 amino acid residues. Additionally, pioglitazone formed other bonding interactions with HIS A:305 and TRP A:59 amino acids (Table 4). The 3D and 2D views of the top three ligand interactions with the *α*-amylase enzyme in the binding site, highlighting non-bond interactions, are presented in Figure 4.

**TABLE 4:** Best top three ligand interactions with the alpha-amylase enzyme based on the binding affinity.

Ligand	Target	No. of HBs	HB residues	HB distance (Å)	Other bond residues
Myricetin-*O*-hexosyl-*O*-hexuronoside	*α*-amylase (4GQR)	09	HIS A:491 ASP A:456 GLN A:7 ARG A:10 LYS A:35 ARG A:392 ARG A:424 GLN A:8 GLY A:36	2.30 2.18 2.25 2.93 2.30 2.87 2.53 3.24 3.20	ILE A:396, VAL A:400
Rutin		06	ASP A:402 ARG A:10 SER A:289 GLN A:8 ARG A:421 THR A:11	2.95 2.97 2.84 2.28 2.76 3.32	PRO A:332
Ursolic acid		01	GLU A:233	2.54	LEU A:162, ALA A:198, TRP A:58, TRP A:59, TYR A:62, HIS A:305
Pioglitazone		02	ASP A:356 TRP A:59	2.00 2.41	HIS A:305, TRP A:59

HB, hydrogen bond.

#### 3.3.3 Top three test ligands and acarbose with α-glucosidase enzyme interactions

**FIGURE 5:**
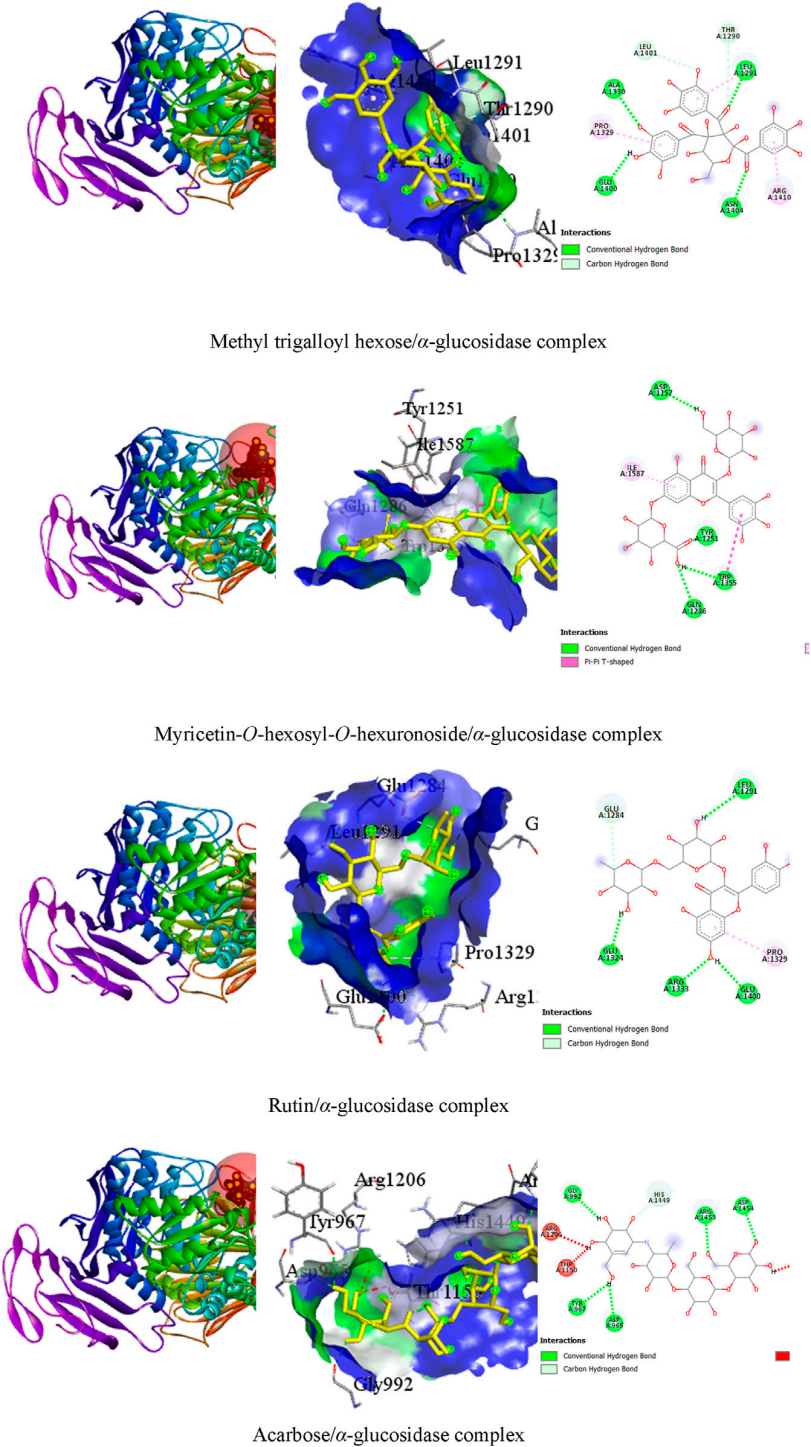
The 3D and 2D views of the top three ligand interactions with the *α*-glucosidase enzyme in the binding site highlight non-bond interactions.

It was observed that the test ligands methyl trigalloyl hexose, myricetin-*O*-hexosyl-*O*-hexuronoside, and rutin formed numerous hydrogen bonds and other bonds with amino acids in the *alpha*-glucosidase enzyme’s binding pocket. Specifically, methyl trigalloyl hexose created six hydrogen bonds with GLU A:1400, LEU A:1291, ALA A:1330, ASN A:1404, THR A:1290, and LEU A:1401 amino acids, along with forming other bonds with PRO A:1329, LEU A:1291, and ARG A:1410 amino acids. Similarly, myricetin-*O*-hexosyl-*O*-hexuronoside formed four hydrogen bonds with TRP A:1355, ASP A:1157, TYR A:1251, and GLN A:1286 amino acids. Additionally, rutin established hydrophobic bonds with TRP A:1355 and ILE A:1587 amino acids in the enzyme’s binding site. Furthermore, rutin showed five hydrogen bonds with GLU A:1324, LEU A:1291, GLU A:1400, ARG A:1333, and GLU A:1284 amino acids and exhibited a hydrophobic bond with the PRO A:1329 amino acid. In contrast, acarbose interacted with the *alpha*-glucosidase enzyme by forming only six hydrogen bonds with ASP A:965, TYR A:967, GLY A:992, ARG A:1453, ASP A:1454, and HIS A:1449 amino acid residues (Table 5). The 3D and 2D views illustrating the top three ligand interactions with the *alpha*-glucosidase enzyme in the binding site, highlighting non-bond interactions, are presented in Figure 5.

**TABLE 5:** Best top three ligand interactions with the α-glucosidase enzyme (based on the binding affinity).

Ligand	Target	No. of HBs	HB residues	HB distance	Other bond residues
Methyl trigalloyl hexose	*α*-glucosidase (3TOP)	06	GLU A:1400 LEU A:1291 ALA A:1330 ASN A:1404 THR A:1290 LEU A:1401	2.48 2.42 2.40 2.17 3.62 2.87	PRO A:1329, LEU A:1291, ARG A:1410
Myricetin-*O*-hexoside-*O*-hexuronoside		04	TRP A:1355 ASP A:1157 TYR A:1251 GLN A:1286	2.46 2.37 1.93 2.77	TRP A:1355, ILE A:1587
Rutin		05	GLU A:1324 LEU A:1291 GLU A:1400 ARG A:1333 GLU A:1284	2.25 2.77 2.40 2.65 3.11	PRO A:1329
Acarbose		06	ASP A:965 TYR A:967 GLY A:992 ARG A:1453 ASP A:1454 HIS A:1449	2.53 2.53 2.22 1.98 2.35 3.57	—

HB, hydrogen bond.

## 4 Conclusion

This study presents the first report on the inhibitory ability of leaf extracts of *T*. *ciliata* and *C. odorata* against *α*-amylase and *α*-glucosidase. Likewise, this research showed a remarkable association between the major identified components of *Cedrela* extracts and *α*-amylase and *α*-glucosidase activity inhibition. The *in silico* molecular docking studies exhibited favorable high binding affinities of the components of *Cedrela* extracts like myricetin-*O*-hexosyl-*O*-hexuronoside, rutin, and ursolic acid with *α*-amylase and *α*-glucosidase. This speculated that these phytoconstituents may significantly contribute to enzyme inhibition activities. Knowing that it is difficult to categorize a single compound responsible for the whole inhibitory activity against these enzymes, we can predict, based on the experimental and *in silico* results, that the *α*-amylase and *α*-glucosidase inhibitory activities of plant extracts are a result of the synergistic outcome of these phytoconstituents, suggesting their anti-diabetic potential. However, our study was restricted to *in vitro* biological investigation of the plant extracts; therefore, the bioavailability assessment and toxicological profile have not been explored. In future studies, these aspects will also be assessed. In addition, additional studies are required for the isolation and identification of individual phytocomponents and assessing their anti-hyperglycemic effect.

## Data Availability

The in silico docking/visualization data presented in this study has been deposited to Any Data via the Figshare partner repository, Digital Identification Number 10.6084/m9.figshare.27641805.
