# Supplementary material for: UPLC-ESI/MSn metabolic profiling of Cedrela odorata L. and Toona ciliata M. Roem and in vitro investigation of their anti-diabetic activity supported with molecular docking studies
Source: Front Chem. 2024 Nov 15;12:1462309. doi: 10.3389/fchem.2024.1462309 (PMC11604428; doi:10.3389/fchem.2024.1462309)
Supplement: Supplementary file 2 [file DataSheet1.docx]

**Figure S1. MS/MS fragmentation pattern for apigenin**

**Figure S2. MS/MS fragmentation pattern for A-type proanthocyanidin dimer**

**Figure S3: MS/MS fragmentation pattern for caffeic acid hexoside**

**Figure S4. MS/MS fragmentation pattern for chicoric acid**

**Figure S5: MS/MS fragmentation pattern for ganolucidic acid B**

**Figure S6: MS/MS fragmentation pattern for icariside I**

**Figure S7: MS/MS fragmentation pattern for kaempferol acetyl-hexoside**

**Figure S8: MS/MS fragmentation pattern for kaempferol-deoxyhexosyl-hexoside**

**Figure S9: MS/MS fragmentation pattern for manniflavone**

**Figure S10: MS/MS fragmentation pattern for methyl trigalloyl hexose**

**Figure S11: MS/MS fragmentation pattern for quercetin-3-*O*-pentoside**

**Figure S12: MS/MS fragmentation pattern for quercetin-*O*-acetyl-hexoside**

**Figure S13: MS/MS fragmentation pattern for rutin**

**Figure S14: MS/MS fragmentation pattern for ursolic acid**
